# Sexual dimorphism, allometric morphological changes, and wild diet of the endemic Antarctic tardigrade *Milnesium rastrum*

**DOI:** 10.1186/s40851-026-00270-x

**Published:** 2026-08-19

**Authors:** Kenta Sugiura, Natsume Takahira, Tomotake Wada

**Affiliations:** 1 Department of Biological Sciences, Graduate School of Science, Tokyo Metropolitan University, Makino Herbarium, Minami-Osawa 1-1, Hachioji, Tokyo, 192-0397 Japan; 2 Department of Polar Science, The Graduate University for Advanced Studies (SOKENDAI), 10-3 Midori-cho, Tachikawa, Tokyo, 190-8518 Japan; 3 Faculty of Environment and Information Sciences, Yokohama National University, 79-7 Tokiwadai, Hodogaya, Yokohama, 240-8501 Kanagawa Japan

**Keywords:** Tardigrades, Antarctica, *Milnesium rastrum*, Sexual dimorphism, Diet

## Abstract

**Background:**

The Antarctic continent harbors unique microfauna that evolved independently under extreme conditions. The tardigrade *Milnesium rastrum*, discovered in East Antarctica, is characterized by atypical, aberrant claw configurations (CC) and pronounced sexual dimorphism. However, its detailed morphology and ecological roles have remained obscure due to an extreme scarcity of specimens. During the 67th Japanese Antarctic Research Expedition (JARE67, 2025–2026), we discovered a moss colony where *M. rastrum* was abundant near Langhovde, providing a rare opportunity for comprehensive morphometric and ecological analyses.

**Results:**

Out of 33 collected individuals, 17 were males, 14 were adult females, and two were juveniles, indicating a balanced sex ratio of approximately 1:1. Significant sexual dimorphism was observed: females were larger (up to 742 μm) than males (up to 549 μm), and males exhibited a more slender posterior body. On leg I, males possessed enlarged, hook-like secondary branches. Males consistently exhibited a {4–4} CC, with a {2–2} pattern on leg I, whereas female body length showed a positive correlation with the number of points on the secondary branches, with larger individuals showing as many as seven points. Intestine content analysis revealed remnants of Hypsibiidae claws and *Diphascon*-like long buccal tubes. This provides direct evidence that *M. rastrum* preys on the sympatric tardigrade *Diphascon langhovdense* in its natural habitat.

**Conclusions:**

This study provides the first comprehensive characterization of sexual dimorphism, growth dynamics, and the wild diet of *M. rastrum*. Our findings clarify distinct morphological variations between sexes and suggest different developmental strategies regarding molting. Additionally, confirming *D. langhovdense* as a natural prey source clarifies the specific ecological role of *M. rastrum* as a predator in the Langhovde microfauna community. Overall, these insights fundamentally advance our understanding of the morphological adaptations and ecological interactions of endemic tardigrades in extreme Antarctic environments.

**Supplementary Information:**

The online version contains supplementary material available at https://doi.org/10.1186/s40851-026-00270-x.

## Background

The Antarctic continent is surrounded by ocean and exposed to one of the harshest natural environments on Earth. Consequently, many species endemic to Antarctica have become established and evolved independently there. This includes the microfauna [1, 2], with endemic species having been discovered among rotifers, nematodes and tardigrades (e.g. the rotifer *Philodina gregaria* Murray, 1910; the nematode *Plectus murrayi* Yeates, 1970; the tardigrade *Acutuncus antarcticus* (Richters, 1904)). However, fundamental questions remain regarding the extent of diversity within these animal groups and the ecological characteristics they exhibit in such harsh conditions.

Tardigrades are organisms found across a wide range of habitats on Earth and of the more than 1,500 species within the phylum Tardigrada [3, 4], 42 species have been described from Antarctica alone [5]. Among species of the genus *Milnesium*, claw configuration (CC [6]), which indicates the number of points on the secondary branches, is typically consistent and used as a taxonomic key. Also, males in 16 species in the genus *Milnesium* exhibit sexual dimorphism (reviewed in [7, 8]), robust hook-shaped claws on the first pair of legs. It has also been reported that males tend to have a slimmer body than females e.g. [9].

In contrast, *Milnesium rastrum* Suzuki, Sugiura, Tsujimoto & McInnes, 2023, which is found only in scattered locations near Lützow-Holm Bay in Dronning Maud Land, East Antarctica, exhibits a unique CC. The number of points on the secondary branches vary irregularly from four to seven among individuals [10]. Although males have also been confirmed, only a single specimen has been found, in the process of molting [10]. Laboratory observations have indicated that this species feeds on rotifers. However, these findings are based on a very small number of specimens. Therefore, critical aspects of its biology remain poorly understood, including sexual dimorphism, allometric morphological changes in CC, and its actual diet in the wild.

From late 2025, we participated in the 67th Japanese Antarctic Research Expedition (JARE67) and collected moss samples near Lützow-Holm Bay. We discovered a colony of *M. rastrum* near Yukidori Valley Hut in Langhovde (Lützow-Holm Bay, Dronning Maud Land, East Antarctica) and conducted a detailed examination of the specimens.

## Methods

### Sample collection

**Fig. 1:**
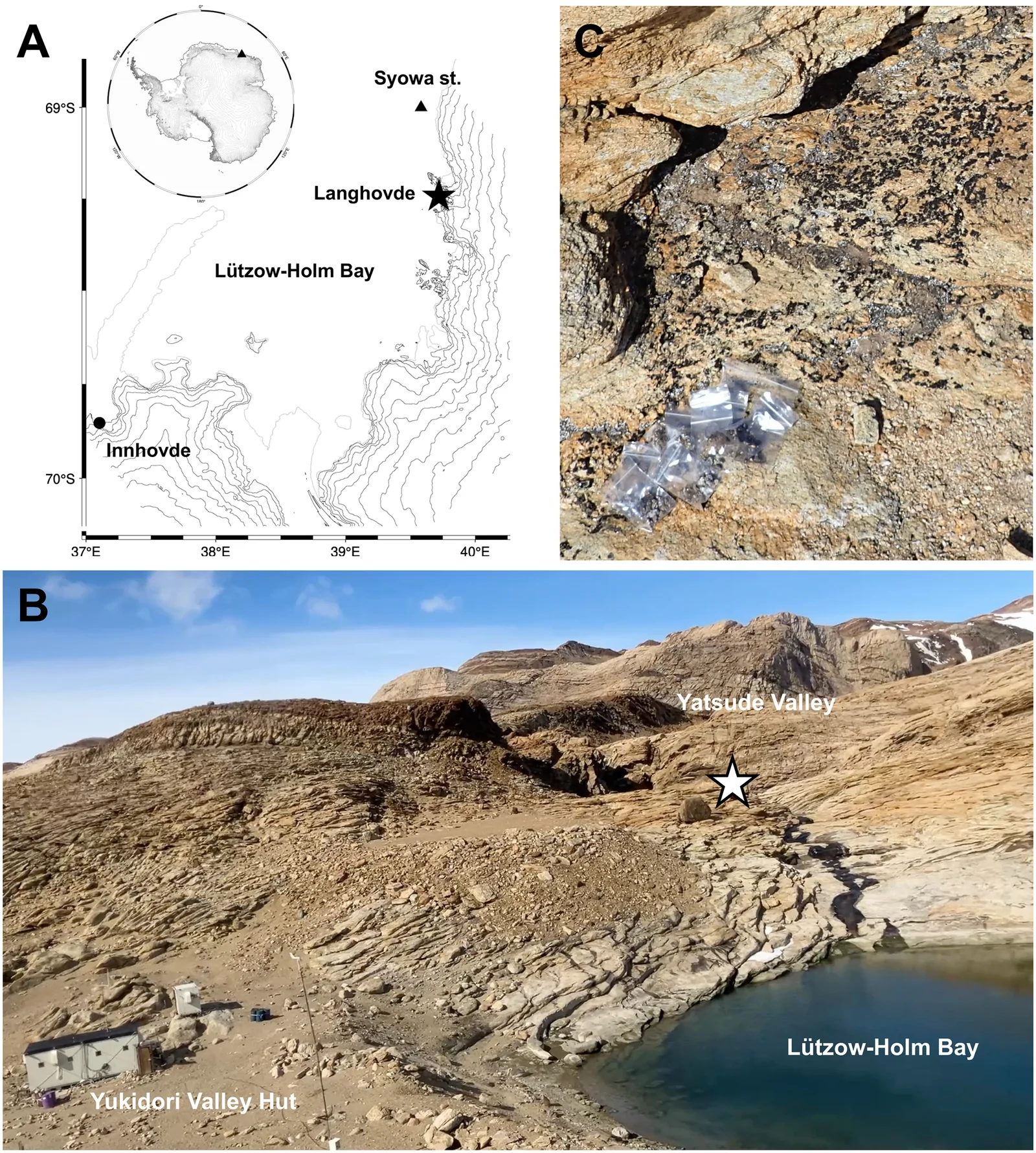
Sampling site in Langhovde, East Antarctica. Maps and pictures of the sampling site in Antarctica. (**A**) Maps showing the sampling site in the Antarctica (top left) and Lützow-Holm Bay. The triangle, star and circle indicate the location of the Syowa Station, the sampling site and Innhovde (the type locality of *M. rastrum*). **B**: a picture of the sampling site. The star indicates the sampling site. **C**: sampled mosses

Moss samples were collected in Langhovde, Lützow-Holm Bay, Dronning Maud Land, East Antarctica (69°14’38.58–68“S 39°43’09.49–54“E, ca.10 a.s.l.) between January 8th to 11th, 2026 (Fig. 1). Moss samples from the surrounding area were also collected. The samples were soaked in water for one day, and were then observed under a stereomicroscope. All *Milnesium* species found were picked, mounted with Hoyer’s medium on glass slides, and sealed with coverslips.

### Morphology observation and morphometrics

**Fig. 2:**
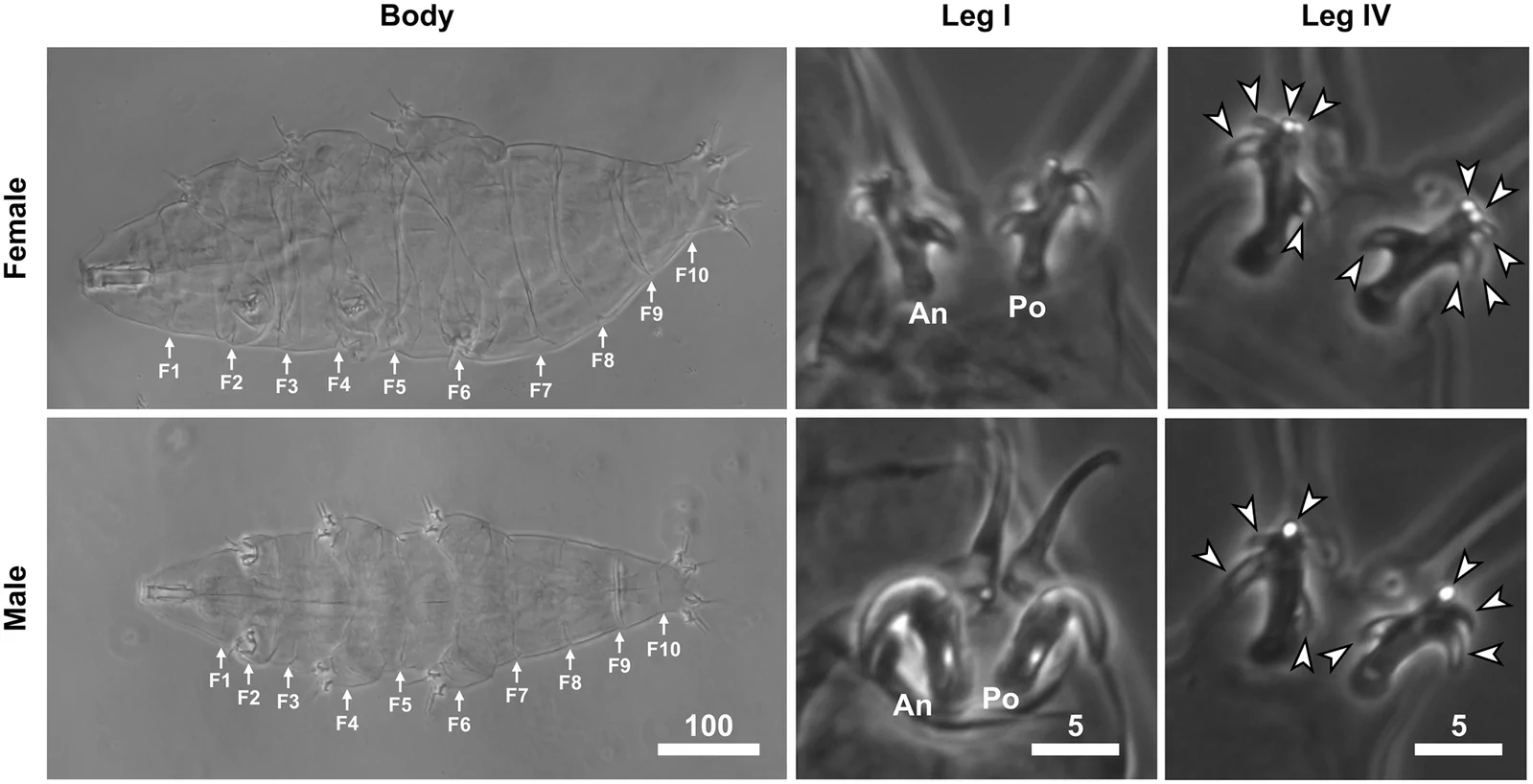
Female and male of *Milnesium rastrum.* specimens of female and male *M. rastrum*. Left: whole body. Center: leg I. Right: leg IV. An and Po are abbreviations of anterior secondary branch and posterior secondary branch, respectively. Arrows indicate body folds of F1 to F10 (‘F’ stands for ‘fold’). Arrowheads indicate points of secondary branches. Scales = μm

The *Milnesium* specimens were observed using phase-contrast microscopy. The structures of the specimens were measured using Apochela v1.4 [11]. The notation for CC followed [12]. The *pt* index (the ratio of measured values to the buccal tube) was used for comparison [13]. Body width was measured at each of the 10 transverse folds (see Fig. 2, abbreviated as F1 to F10). To compare the samples, a score was calculated by dividing body width by body length. To compare CC, the average number of points per leg across each secondary branch was calculated. Measurements were performed using ImageJ 1.53k (https://imagej.nih.gov/ij/) based on photographs. A Wilcoxon rank-sum test was used to statistically compare the claw configurations of males and females, while a Student’s t-test was used to compare body length, secondary branch length and body width on R v.3.6.1 [14].

Sexual dimorphism was quantified using the two-step ratio Sexual dimorphism index (SDI [15]). For each morphological trait, the SDI was calculated for every possible female and male pair. The resulting SDI values were then averaged to obtain a single estimate for the trait, in accordance with the procedure outlined for Echiniscidae tardigrades [16]. Positive SDI values indicate female-biased dimorphism, whereas negative values indicate male-biased dimorphism; the absolute value represents the magnitude of dimorphism. Statistical significance was assessed using a permutation test in which sex labels were randomly reassigned among individuals while preserving the original sample sizes (500 permutations). The observed SDI was then compared with the null distribution to obtain a two-tailed, permutation-based *p*-value. As multiple morphological traits were analyzed, the permutation-derived *p*-values were adjusted using the Benjamini–Hochberg false discovery rate (FDR) procedure. Traits with an FDR-adjusted permutation *p*-value < 0.05 were considered to exhibit significant sexual dimorphism. R v3.6.1 was used for comparisons [14]. The code used in this study was uploaded on FigShare (10.6084/m9.figshare.32843480).

## Results

### Sexual dimorphism of *Milnesium rastrum*

**Fig. 3:**
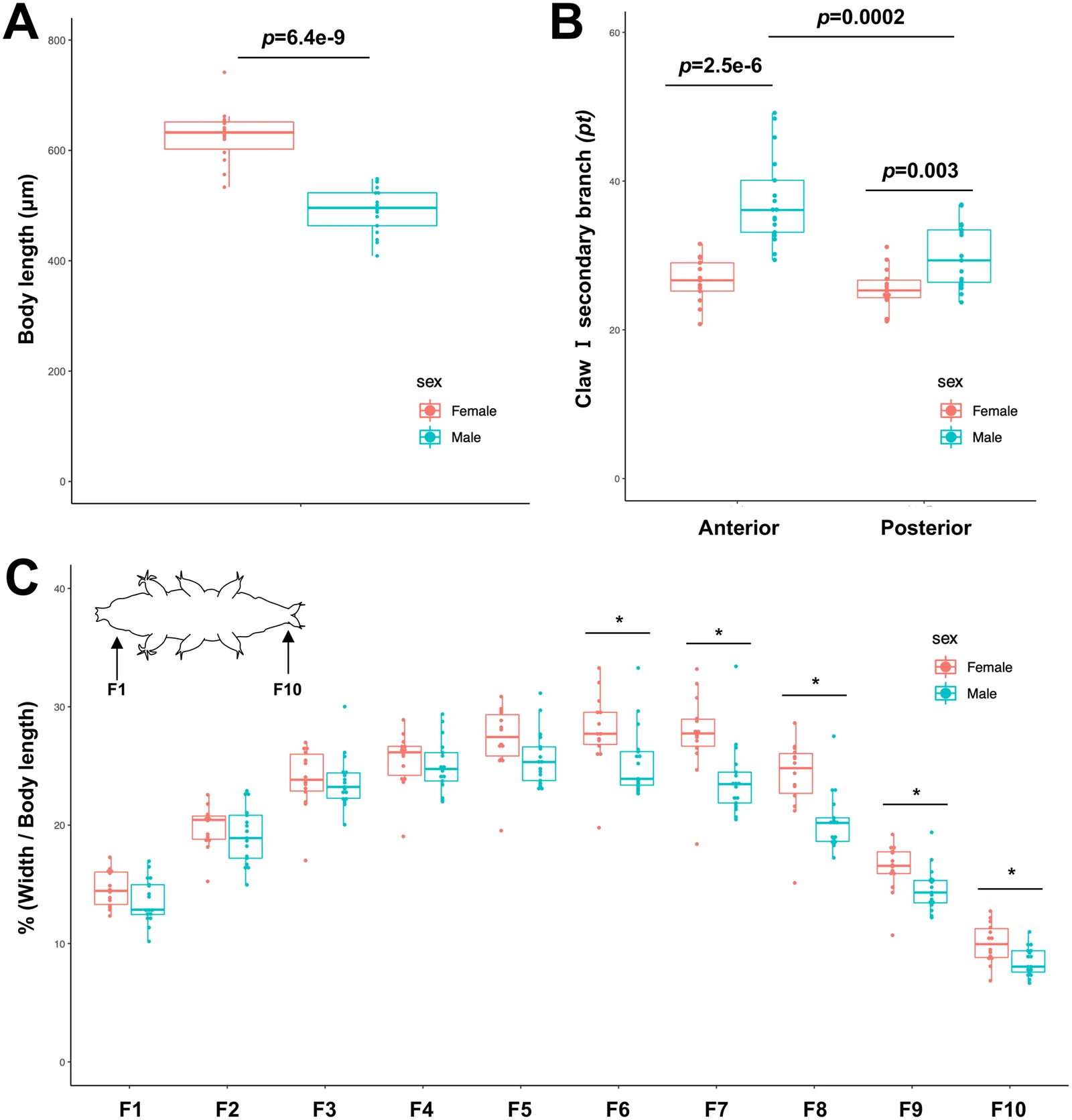
Morphometrical comparison between female and male. **A**: body length (μm). *p*-value is indicated. **B**: anterior and posterior secondary branch length (*pt* index) of leg I. *p*-values with significant differences are indicated (Student’s t-test). **C**: body width (%body length). ‘F’ stands for ‘fold’. 25th to 75th percentiles with a line at the median. Whiskers extend to 1.5 times the interquartile range. Individual dots indicate respective values. *: *p* < 0.05 (Student’s t-test)

At Langhovde, near the Yukidori Valley Hut, we discovered that a moss colony located directly behind a glacial erratic serves as a colony where *M. rastrum* lives in abundance (Fig. 1). All 33 individuals collected during field observations were mounted as slide specimens (Fig. 2). Of these, 17 were males, 14 were adult females, and two were juveniles. Morphometrical data were on Tables 1, 2, and Table S1. The specimens of *M. rastrum* possessed secondary branches with up to seven points (Fig. 2, see next section). While the males measured up to 549 μm, some females were found to be as large as 742 μm (Tables 1 and 2, Table S1). Females were significantly longer in body length (*p* < 0.01 in a Student’s t-test, Fig. 3A). In male specimens, the secondary branch of leg I displayed the robust hook shape and, when compared using *pt* values, was significantly larger than that of females (Fig. 3B). Furthermore, when comparing the sizes of the anterior and posterior secondary branches of the male’s leg I, the anterior secondary branch was significantly larger (Fig. 3B). In addition, we measured the body width at 10 body folds and divided these values by body length to determine the slenderness of the body relative to its length (see Fig. 2). A comparison between males and females revealed that, in the region posterior to the sixth to tenth body fold (roughly posterior to the third leg), males were more slender than females (Fig. 3C, *p* < 0.05). Analysis using SDI revealed sex differences in buccal tube width (both actual measurements and *pt* values) and the lengths of several claws (*pt* values), in addition to the traits mentioned above (Table S2). Specifically, females had wider buccal tubes, while males often had longer secondary branches.

**Table 1:** Morphometrics of female *Milnesium rastrum*

Character	N	Range						Mean		SD	
		µm			*pt*			µm	*pt*	µm	*pt*
Body length	14	533.4	–	741.6	*993.2*	–	*1419.8*	627.1	*1239.6*	50.6	*115.4*
Peribuccal papillae length	6	2.4	–	6.2	*5.2*	–	*10.9*	3.9	*7.2*	1.4	*2.2*
Lateral papillae length	6	3.7	–	7.8	*8.5*	–	*13.6*	5.1	*10.3*	1.4	*1.8*
Buccal tube											
Length	14	41.7	–	65.3		–		51.2	–	7.6	–
Stylet support insertion point	14	23.2	–	45.8	*53.6*	–	*73.5*	30.5	*59.5*	6.2	*5.3*
Anterior width	14	11.1	–	23.7	*22.5*	–	*36.3*	14.8	*28.9*	3.2	*4.0*
Standard width	14	10.3	–	18.8	*20.4*	–	*37.5*	14.0	*27.4*	3.1	*4.9*
Posterior width	14	6.2	–	17.1	*14.8*	–	*34.4*	12.0	*23.3*	3.1	*4.9*
Posterior/anterior width ratio	14	0.5	–	1.1		–		0.8	–	0.2	–
Claw I heights											
Posterior (Posterior (External)) primary branch	14	13.8	–	22.6	*27.9*	–	*45.0*	17.7	*34.9*	2.6	*4.4*
Posterior (External) base + secondary branch	14	9.9	–	15.8	*21.2*	–	*31.1*	13.0	*25.6*	2.0	*2.7*
Posterior (External) spur	14	1.3	–	3.8	*3.2*	–	*5.9*	2.2	*4.3*	0.7	*0.9*
Posterior (External) branches length ratio	14	0.6	–	0.9		–		0.7	–	0.1	–
Anterior (Internal) primary branch	14	10.7	–	25.8	*22.7*	–	*45.2*	16.8	*33.1*	3.7	*6.8*
Anterior (Internal) base + secondary branch	13	10.3	–	15.7	*20.8*	–	*31.6*	13.3	*26.6*	1.7	*3.1*
Anterior (Internal) spur	13	1.2	–	4.4	*2.5*	–	*10.5*	2.5	*5.1*	1.1	*2.2*
Anterior (Internal) branches length ratio	13	0.5	–	1.2		–		0.8	–	0.2	–
Claw II heights											
Posterior (External) primary branch	14	15.6	–	26.4	*25.3*	–	*50.8*	20.1	*39.7*	3.9	*7.1*
Posterior (External) base + secondary branch	13	9.8	–	15.1	*17.7*	–	*30.6*	12.8	*25.7*	1.6	*3.8*
Posterior (External) spur	13	1.0	–	3.9	*2.5*	–	*7.8*	2.4	*4.8*	0.9	*1.7*
Posterior (External) branches length ratio	13	0.5	–	0.8		–		0.6	–	0.1	–
Anterior (Internal) primary branch	14	14.0	–	29.1	*27.4*	–	*50.9*	18.9	*37.2*	4.2	*7.1*
Anterior (Internal) base + secondary branch	14	9.6	–	15.7	*19.1*	–	*29.7*	12.5	*24.7*	1.6	*3.5*
Anterior (Internal) spur	14	1.0	–	4.5	*2.5*	–	*8.6*	2.9	*5.8*	0.9	*1.9*
Anterior (Internal) branches length ratio	14	0.5	–	0.8		–		0.7	–	0.1	–
Claw III heights											
Posterior (External) primary branch	14	12.9	–	25.4	*31.0*	–	*50.6*	21.0	*41.3*	3.2	*6.1*
Posterior (External) base + secondary branch	14	10.0	–	15.7	*23.0*	–	*30.8*	13.7	*26.9*	1.7	*2.1*
Posterior (External) spur	14	1.6	–	4.0	*3.4*	–	*6.9*	2.6	*5.1*	0.7	*1.1*
Posterior (External) branches length ratio	14	0.5	–	0.9		–		0.7	–	0.1	–
Anterior (Internal) primary branch	14	14.8	–	23.7	*31.1*	–	*50.4*	19.5	*38.3*	3.0	*5.5*
Anterior (Internal) base + secondary branch	13	10.3	–	15.6	*22.0*	–	*31.0*	13.3	*26.7*	1.6	*2.8*
Anterior (Internal) spur	13	1.5	–	5.9	*3.2*	–	*10.4*	3.2	*6.2*	1.2	*1.9*
Anterior (Internal) branches length ratio	13	0.6	–	0.9		–		0.7	–	0.1	–
Claw IV heights											
Anterior primary branch	14	18.3	–	31.0	*37.2*	–	*54.6*	23.1	*45.4*	3.7	*5.4*
Anterior base + secondary branch	14	9.3	–	17.6	*18.8*	–	*32.1*	14.0	*27.5*	2.4	*3.6*
Anterior spur	14	1.2	–	5.4	*2.6*	–	*11.4*	2.7	*5.2*	1.2	*2.4*
Anterior branches length ratio	14	0.5	–	0.8		–		0.6	–	0.1	–
Posterior primary branch	14	15.8	–	29.5	*32.0*	–	*54.3*	21.9	*43.0*	4.2	*6.7*
Posterior base + secondary branch	14	11.8	–	18.3	*26.1*	–	*37.4*	15.1	*29.6*	2.2	*3.4*
Posterior spur	14	1.0	–	4.6	*2.3*	–	*9.7*	3.3	*6.4*	1.1	*1.9*
Posterior branches length ratio	14	0.5	–	0.9		–		0.7	–	0.1	–

**Table 2:** Morphometrics in male *Milnesium rastrum*

Character	N	Range						Mean		SD	
		µm			*pt*			µm	*pt*	µm	*pt*
Body length	17	408.9	–	548.5	*914.1*	–	*1581.8*	492.5	*1176.8*	42.1	*180.7*
Peribuccal papillae length	12	2.5	–	3.4	*5.6*	–	*8.3*	23.8	*52.8*	72.4	*159.2*
Lateral papillae length	15	2.4	–	5.2	*5.3*	–	*15.0*	4.1	*9.9*	2.4	*5.3*
Buccal tube											
Length	17	33.3	–	48.7		–		42.4	–	4.7	–
Stylet support insertion point	17	19.2	–	36.0	*42.1*	–	*77.0*	23.9	*56.6*	5.2	*10.7*
Anterior width	17	9.0	–	13.5	*19.7*	–	*30.9*	10.3	*24.6*	1.2	*3.7*
Standard width	17	7.6	–	11.4	*17.3*	–	*25.7*	8.7	*20.8*	1.0	*3.0*
Posterior width	17	6.6	–	9.9	*14.8*	–	*24.0*	8.0	*19.1*	0.9	*2.2*
Posterior/anterior width ratio	16	0.6	–	1.1		–		0.8	–	0.1	–
Claw I heights											
Posterior (Posterior (External)) primary branch	17	9.3	–	18.4	*26.8*	–	*46.3*	15.4	*36.4*	2.0	*5.2*
Posterior (External) base + secondary branch	17	8.9	–	16.7	*23.7*	–	*36.8*	12.6	*29.9*	2.0	*4.3*
Posterior (External) spur	17	1.0	–	3.1	*2.5*	–	*8.3*	1.9	*4.6*	0.6	*1.5*
Posterior (External) branches length ratio	16	0.6	–	1.0		–		0.8	–	0.1	–
Anterior (Internal) primary branch	17	13.0	–	18.6	*28.8*	–	*47.4*	16.0	*38.1*	1.6	*5.6*
Anterior (Internal) base + secondary branch	17	13.0	–	19.0	*29.4*	–	*49.2*	15.6	*37.4*	1.7	*6.0*
Anterior (Internal) spur	17	1.2	–	2.9	*2.9*	–	*8.8*	1.8	*4.3*	0.4	*1.3*
Anterior (Internal) branches length ratio	16	0.8	–	1.1		–		1.0	–	0.1	–
Claw II heights											
Posterior (External) primary branch	17	13.8	–	23.8	*36.7*	–	*66.7*	19.7	*47.0*	2.5	*8.1*
Posterior (External) base + secondary branch	16	8.2	–	13.8	*18.1*	–	*37.1*	11.8	*28.2*	1.3	*4.9*
Posterior (External) spur	16	1.2	–	3.2	*2.9*	–	*7.5*	2.2	*5.3*	0.6	*1.4*
Posterior (External) branches length ratio	15	0.5	–	0.8		–		0.6	–	0.1	–
Anterior (Internal) primary branch	17	14.6	–	23.5	*33.7*	–	*65.7*	19.6	*46.7*	2.6	*8.4*
Anterior (Internal) base + secondary branch	17	10.4	–	14.9	*23.3*	–	*38.6*	12.5	*29.8*	1.3	*4.3*
Anterior (Internal) spur	17	1.2	–	4.2	*2.7*	–	*9.4*	2.8	*6.5*	0.9	*2.0*
Anterior (Internal) branches length ratio	16	0.5	–	0.8		–		0.6	–	0.1	–
Claw III heights											
Posterior (External) primary branch	17	16.7	–	23.7	*38.2*	–	*59.6*	20.4	*48.5*	1.9	*5.4*
Posterior (External) base + secondary branch	16	7.9	–	14.3	*22.8*	–	*37.9*	12.2	*29.0*	1.6	*4.3*
Posterior (External) spur	16	1.2	–	2.7	*2.6*	–	*6.4*	2.0	*4.8*	0.4	*1.1*
Posterior (External) branches length ratio	15	0.5	–	0.7		–		0.6	–	0.1	–
Anterior (Internal) primary branch	17	15.5	–	23.6	*34.6*	–	*60.3*	19.6	*46.6*	2.6	*6.7*
Anterior (Internal) base + secondary branch	17	9.4	–	14.6	*20.7*	–	*38.1*	12.2	*29.2*	1.6	*4.7*
Anterior (Internal) spur	17	1.3	–	3.8	*3.1*	–	*8.2*	2.5	*5.8*	0.7	*1.5*
Anterior (Internal) branches length ratio	16	0.5	–	0.8		–		0.6	–	0.1	–
Claw IV heights											
Anterior primary branch	17	15.1	–	24.8	*33.7*	–	*69.4*	21.0	*50.3*	2.6	*9.7*
Anterior base + secondary branch	16	2.5	–	15.3	*5.4*	–	*40.7*	12.7	*30.5*	3.1	*8.3*
Anterior spur	16	1.2	–	20.4	*3.0*	–	*43.6*	3.9	*8.9*	4.5	*9.4*
Anterior branches length ratio	15	0.1	–	0.8		–		0.6	–	0.1	–
Posterior primary branch	17	11.4	–	25.7	*25.4*	–	*69.0*	21.4	*51.2*	3.5	*10.7*
Posterior base + secondary branch	16	9.9	–	15.7	*22.2*	–	*43.5*	13.6	*32.4*	1.5	*5.1*
Posterior spur	16	1.6	–	4.4	*3.6*	–	*11.6*	2.8	*6.7*	0.8	*2.0*
Posterior branches length ratio	16	0.5	–	0.9		–		0.6	–	0.1	–

### Claw configuration aberration of *Milnesium rastrum*

**Fig. 4:**
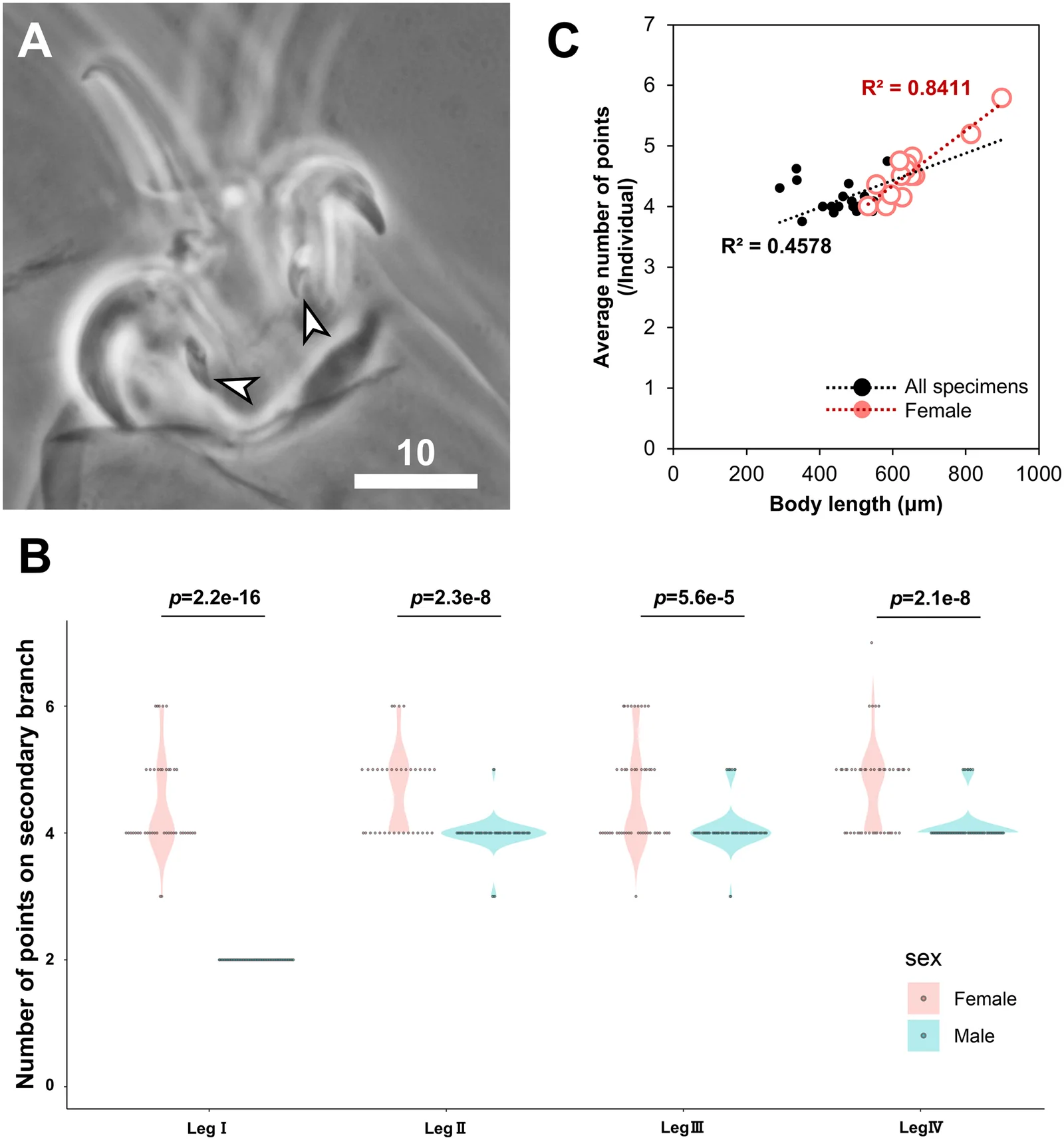
Claw configuration of female and male.** A**: spurs of male secondary branch. Scale = μm. **B**: averaged number of points on secondary branches. Individual dots indicate respective values. *p*-values with significant differences are indicated (Wilcoxon rank-sum test). **C**: correlation between average number of points per secondary branch per leg and body length. Data includes described in Suzuki et al. [10]. “All specimens” includes morphometrical data of hatchlings, juveniles, males, and females. Correlation coefficients R^2^ values are indicated.

At the base of the male’s secondary branches on leg I of *M. rastrum*, there were spurs similar to those found on the other secondary branches (Fig. 4A). Therefore, we determined that the CC of leg I was {2–2}. All males exhibited the {2–2} pattern on leg I. We counted the number of points on the secondary branches of both males and females and calculated the average number of points per secondary branch per leg (Fig. 4B). Leg I in males was consistently limited to two points, whilst legs 2–4 generally had four points. In contrast, the median number of points for females across all legs was 4–5, and some individuals were observed to have as many as seven. Including data from the paper by Suzuki et al. [10], we examined the correlation between body length and the average number of points per secondary branch per leg (Fig. 4C). In the dataset encompassing hatchlings, juveniles, males, and females, the correlation coefficient was 0.46, indicating a weak correlation. In contrast, the correlation coefficient for the data comprising only females was 0.84, indicating a strong correlation.

### Diet of *Milnesium rastrum*

**Fig. 5:**
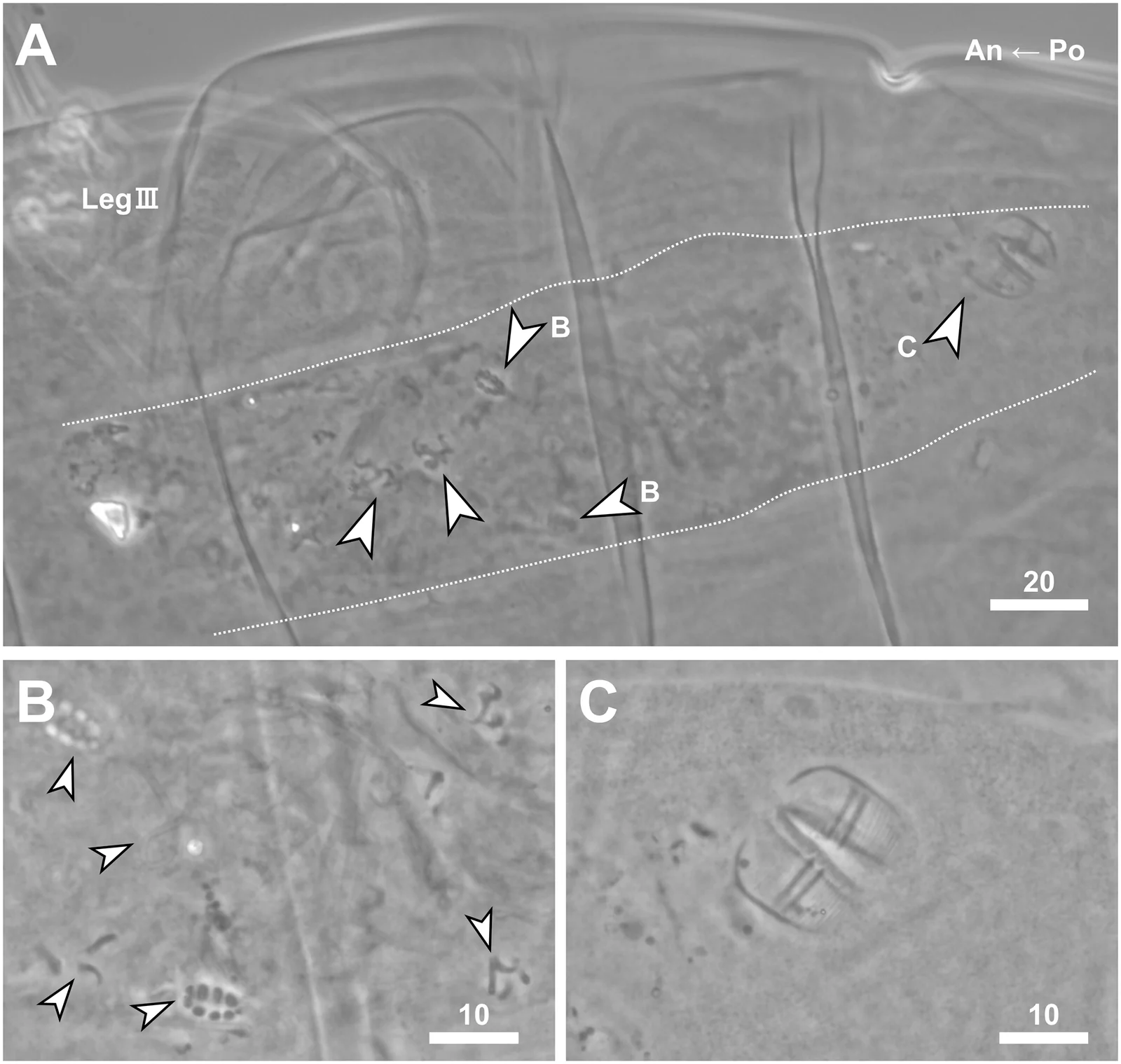
Intestine of *Milnesium rastrum.* **A**: intestine of *M. rastrum* specimen. An and Po indicate anterior and posterior, respectively. Arrowheads indicate gastric contents. Dotted lines indicate the outline of the intestine. Expanded images of labeled B and C region are shown in lower panels. **B**: tardigrades in the intestine. Arrowheads indicate claws, buccal tubes, or placoids. **C**: a trophi of rotifer mastax in the intestine. Scales = μm

We examined the intestinal contents of the specimens (Fig. 5), and frequently found the placoids, buccal tubes, and claws of other tardigrades (Fig. 5B), as well as trophi of bdelloid rotifer mastaces (Fig. 5C). It was not uncommon to find fragments of multiple tardigrades within both female and male individuals, indicating that *M. rastrum* preys on other tardigrades in its wild habitat. The tardigrade fragments found in the intestine had claws with a Hypsibiidae-type, and traces resembling four placoids associated with pharynx were also observed (Fig. 5B). Furthermore, *Diphascon-*like long buccal tubes attached four placoids were found in the intestine (Fig. 5B).

## Discussion

### Geographical distribution of *Milnesium rastrum*

This report marks the fourth record of a species believed to be *M. rastrum*. Sudzuki reported a single specimen from Langhovde in 1964 [17], followed by Dastych in 1984 [18], and finally, Suzuki et al. described the species in 2023 [10]. Although the exact location within Langhovde where Sudzuki collected his specimen is unclear, it is certain that it was not far from the site of the present collection [17]. Dastych’s report states that the specimen was collected near Molodeznaya [18], while Suzuki et al. designated Innhovde as the type locality for this species [10]. Molodeznaya, Langhovde, and Innhovde are approximately 300–450 km apart, and the species is likely scattered throughout this region, at least.

### Sexual differences of *Milnesium rastrum*

The original description [10] was based on a limited number of specimens, and the only male specimen was an individual undergoing the molt to adulthood. In this study, the discovery of an area where numerous *M. rastrum* specimens could be collected enabled access to more detailed morphological data. Statistically, it was first confirmed that males are smaller and more slender than females. One possible explanation is that females accommodate developing ovaries. SDI analysis indicated that the buccal tube is thicker in females. On the other hand, fragments of tardigrades and trophi of rotifers were found in both males and females; therefore, based on the current data, it is not possible to assess whether differences in feeding habits are related to buccal tube thickness in *M. rastrum*. Furthermore, this study confirmed a positive correlation between female body size and the number of points on the secondary branches. If *M. rastrum* tends to increase in body size with each molt and the number of points also increase, this would be consistent with the observations of this study: that females have more points than males. The CC of males prior to sexual maturity is of the typical “juvenile type” [10]. It is possible that, in males, the number of points and the body length do not change after the molting into adulthood.

Among the 58 currently described species in the genus *Milnesium* [4], males have been reported in 16 species (Table 3), and in all of them, the secondary branch of leg I is highly developed in a hook-like manner. If this structure were necessary for walking, it would not be developed exclusively in males; nor, given the shortness of the legs themselves, is it likely used in combat between males. *Pseudobiotus megalonyx* Thulin, 1928, which, like the milnesiids, belongs to the class Eutardigrada, lays its eggs in molted exuvia, similar to the milnesiids, and the claws on the males’ forelegs differ from those of the females (reviewed in [7, 8]. *Pseudobiotus megalonyx* reproduces sexually, and there are descriptions of males boring holes into the molted exuvia where females are laying eggs in order to ejaculate (reviewed in [35]). Based on these findings, we hypothesize that these hook-like claws are used to restrain the female during mating or to create holes for ejaculation. Although no observations of mating behavior in milnesiids have been reported to date, we look forward to future research in this area.

**Table 3:** Sex ratio of *Milnesium* species including specimens of Suzuki et al., 2023 [10]

	Male	Female	%Male	References
*M. inceptum*	Very few	Parthenogenetic	-	[9]
*Milnesium* sp.	1	25	3.8	[19]
*Milnesium* sp.	2	36	5.3	[20]
*M. milenae*	1	8	11.1	[21]
*M. grandicupula*	3	22	12.0	[22]
*M. tetralamellatum*	1	6	14.3	[23]
*M. beasleyi*	1	5	16.7	[24]
*M. eurystomum*	17	84	16.8	[25]
*M. lagniappe*	3	12	20.0	[26]
*M. warrenwilsonensis*	5	20	20.0	[27]
*M. dornensis*	10	37	21.3	[28]
*M. fridae*	5	16	23.8	[29]
*Milnesium* sp.	18	57	24.0	[20]
*M. matheusi*	3	9	25.0	[30]
*M. myriae*	1	3	25.0	[21]
*Milnesium* sp. MilnC	1	2	33.3	[31]
*Milnesium* sp.	9	17	34.6	[20]
*Milnesium* sp.	29	47	38.2	[20]
*M. decorum*	5	5	50.0	[32]
*M. swansoni*	5	5	50.0	[33]
***M. rastrum***	**18**	**17**	**51.4**	[10]**; This study**
*M. burgessi*	?	?	-	[34]

### Sex ratio of the genus *Milnesium*

Furthermore, this study revealed that males are present in *M. rastrum*, and the sex ratio is approximately 1:1. Of the 21 dioecious species or populations in the genus *Milnesium*, 18 have a higher proportion of females (see Table 3). The remaining three species—*Milnesium decorum* Morek, Wałach & Michalczyk, 2022 [32], *Milnesium swansoni* Young, Chappell, Miller & Lowman, 2016 [33], and *M. rastrum*—show a sex ratio close to 1:1. On the other hand, a major issue is the small number of specimens. As with *Milnesium eurystomum* Maucci, 1901, a survey involving at least 100 specimens would be necessary [25]. Imbalances in sex ratios have been reported in many tardigrade species outside the milnesiids as well (e.g. [36–39]). To date, no sex chromosomes have been found in tardigrades, and it has been reported that no sex-specific regions can be detected even through comparisons of male and female genomes [40]. Elucidating the sex-determination system in tardigrades is desirable in order to understand the causes of sex ratio variation.

### Ecological relation between *Milnesium rastrum* and *Diphascon langhovdense*

Although Suzuki et al. reported finding a rotifer trophi within the intestine of *M. rastrum* [10], these were fed to the organisms under laboratory conditions. Instances of milnesiids preying on other tardigrades have been reported in e.g. *Milnesium* sp. [41], *Milnesium burgessi* Schlabach, Donaldson, Hobelman, Miller & Lowman, 2018 [34], *Milnesium lagniappe* Meyer, Hinton & Dupré, 2013 [26, 42], and *M. eurystomum* [25], and this phenomenon is by no means rare. In particular, McInnes et al. reported the discovery of *Diphascon* sp. with a long buccal tube in the intestine of a milnesiid from Antarctica [41]. Given the presence of Hypsibiidae claws, the four placoids (three macroplacoids and one microplacoid), and the shape of buccal tube remnants in the intestine of *M. rastrum* is reasonable to conclude that they belong to *Diphascon langhovdense* Sudzuki, 1964, which was also found with *M. rastrum* in Langhovde [17]. This observation indicated that *M. rastrum* actively preys on *D. langhovdense* in the natural environment. *Acutuncus antarcticus* is found across a wide area of Antarctica, including Langhovde [43]. In the specimens examined in this study, no short buccal tubes resembling those of *A. antarcticus* were found within the intestines. One factor worth noting may be the body size of the prey. Adult *A. antarcticus* exceeds 300 µm [44], whereas *D. langhovdense* is just over 100 µm [17]. *Milnesium rastrum* is probably capable of swallowing at least small prey whole. Detailed examinations of the intestines of *M. rastrum* collected from another location would likely clarify whether *M. rastrum* preferentially preys on *D. langhovdense* or whether it feeds indiscriminately on other species, such as *A. antarcticus*.

## Conclusions

In conclusion, our investigation of the *M. rastrum* colony at Langhovde significantly advances the morphological and ecological understanding of this endemic Antarctic tardigrade. We successfully documented pronounced sexual dimorphism, establishing that males are smaller and more slender than females, and possess highly specialized, enlarged robust hook-like claws on their first pair of legs. The strong positive correlation between female body length and the number of points on the secondary branches suggests that CC may change with body growth in females. Furthermore, it was suggested that the sex ratio of *M. rastrum* in the wild is approximately 1:1. Finally, the identification of *D. langhovdense* remnants within the intestine confirms that *M. rastrum* is a predator of other tardigrades.

## Electronic supplementary material

Below is the link to the electronic supplementary material.


Supplementary Material 1



Supplementary Material 2


## Data Availability

Draft data of this study is uploaded on FigShare (DOI: 10.6084/m9.figshare.32843480).
